# Fluid pathways identified beneath Narlı Lake (Central Anatolia) show the geothermal potential of former volcanoes

**DOI:** 10.1038/s41598-021-87743-5

**Published:** 2021-04-22

**Authors:** Maren Brehme, Ronny Giese, Uğur Erdem Dokuz, Fatih Bulut

**Affiliations:** 1 Department of Geoscience and Engineering, Delft University of Technology, Stevinweg 1, 2628CN Delft, The Netherlands; 2 Helmholtz Centre Potsdam, GFZ German Research Centre for Geosciences, Geoenergy, Telegrafenberg, 14473 Potsdam, Germany; 3 Department of Geological Engineering, Faculty of Engineering, Niğde Ömer Halisdemir University, 51240 Niğde, Turkey; 4 Geodesy Department, Kandilli Observatory and Earthquake Research Institute, Boğaziçi University, Uskudar, 34684 Istanbul, Turkey

**Keywords:** Structural geology, Geochemistry, Geology, Tectonics

## Abstract

We investigated the volcanic Narlı Lake in Central Anatolia combining high-resolution bathymetry and geochemical measurements. In this study, we present it as proof of a new concept to verify fluid pathways beneath lakes integrating the structure of the geothermal reservoir into the surrounding tectonic frame. We recognized dextral faults fracturing inherited volcanic formations and thus generating highly permeable zones beneath the lake. At intersection points of faults, reservoir fluids discharge from deep holes as imaged by the high-resolution bathymetry at the bottom of the Narlı Lake. Onshore, the tectonic setting also generates both extensional and compressional structures. Extensional structures result in extensive fluid discharge through hot springs while compressional structures do not discharge any fluid. The water of the lake as well as in the hot springs is highly saline and has relatively high concentrations of Cl, HCO_3_, SO_4_, Na, Ca, Mg, and Si. In several hot springs, we observed mixtures of high-saline fluids having a deep origin and low-saline shallow groundwater. We observed discharge into the lake by gas bubbles, which contain probably CO_2_ or H_2_S. Mineral precipitation indicates a carbonatic source at the lake bottom and along the shoreline. Extensive travertine precipitation also occurs near hot springs along the nearby extensional zone of Ihlara Valley. In summary, the composition of fluids and minerals is controlled by water–rock interaction through the volcanic and carbonatic rocks beneath this volcanic lake.

## Introduction

Physical and chemical properties of volcanic lakes play a key role in understanding fluid-pathways underneath^1^. Fluids discharging from underlying reservoirs into the lake have recently been observed through pockmarks and identified as sweet-spots in drilling for geo-resources^1^. These pockmarks and faults are visible in the topographic structure of the lake bottom. They are better preserved than in onshore outcrops, due to reduced erosion rates in the aquatic environment.

**Figure 1:**
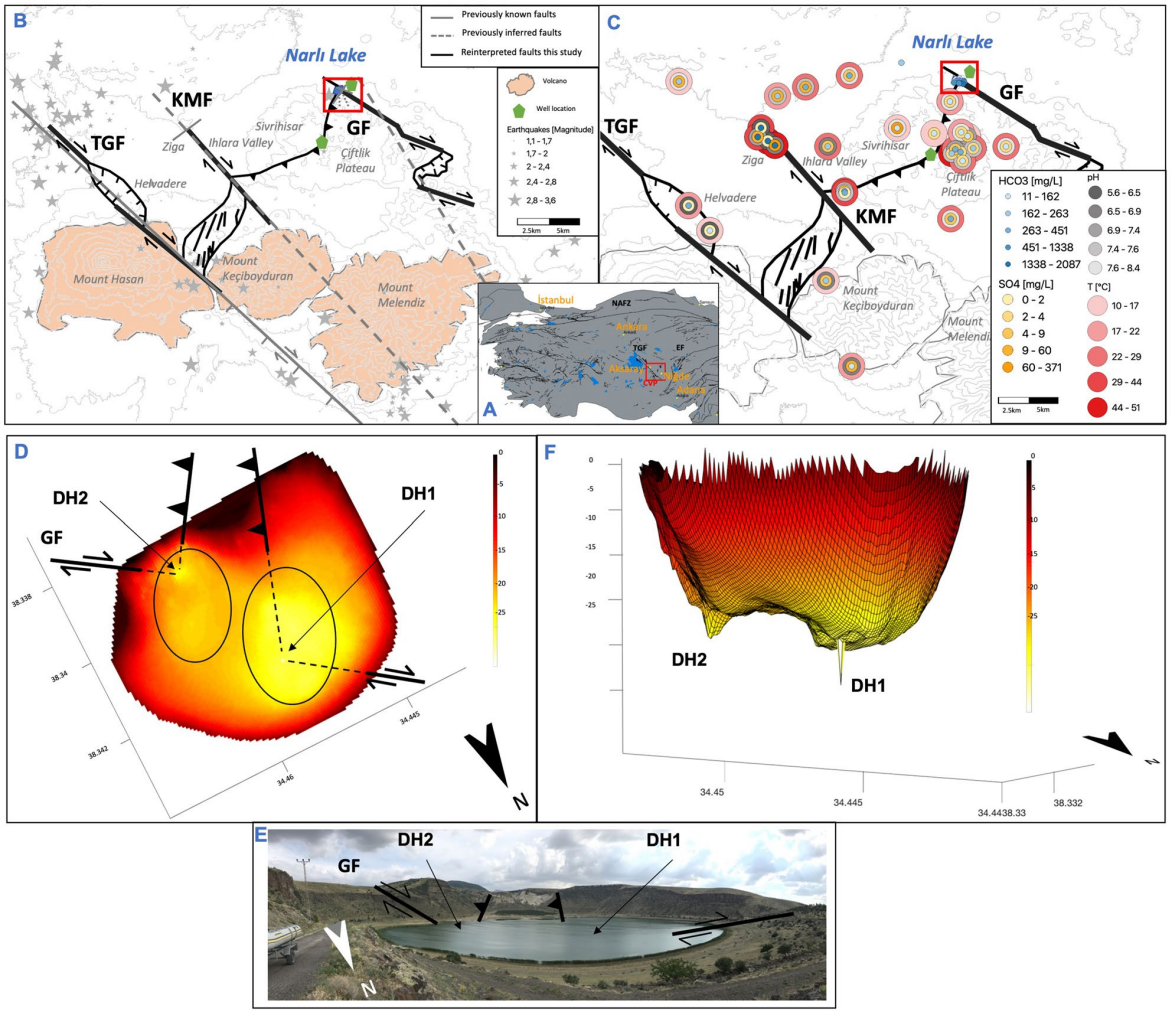
(**A**) Location of study area in Central Anatolia, CVP = Cappadocia volcanic province, TGF = Tuz Gölü Fault, EF = Ecemiş Fault, NAFZ = North Anatolian Fault Zon^32^). (**B**) Known and inferred faults from Dhont et al.^8^, Toprak and Göncüoglu^9^, Gevrek and Kazanci^10^, Afsin et al.^11^ together with reinterpreted faults from this study, KMF = Keçiboyduran-Melendiz Fault, GF = Göllüdağ Fault. Earthquake locations from KOERI-RETMC (2020). (**C**) Location and hydrochemical properties of hot springs with tectonic setting (properties from Burcak^12^ and Şener et al.^2^ and from this study). Red box indicates (**D**). All maps were drawn using QGIS software version 3.12, http://www.qgis.org (**D**) Bathymetry map of Narlı Lake with location of deep holes (DH1, DH2) and faults, including their type, and photo of Narlı Lake (**E**), (**F**) 3D bathymetry with deep holes DH1 and DH2.

In this study, we apply a formerly presented method^1^ to outline the geothermal potential in the Cappadocia Volcanic Province, NW of Niğde in Turkey (CVP hereafter) (Fig. 1). This area hosts the hottest geothermal well of Turkey (295 °C at 3850 m), only 10 km S of the volcanic Narlı Lake (3S Kale) (Fig. 1B).

### Site description

The CVP is located between the cities of Aksaray and Niğde (Fig. 1) on the Central Anatolian plateau at 1200 to 3300 mas. Tectonically, it is N of the junction between the large Tuz Gölü Fault and Ecemiş Fault (TGF and EF in Fig. 1A). Narlı Lake is located at the N edge of the study area and ca. 600 × 900 m large. The crater lake is known for its healing warm water for health treatment in recreation centers. Previous tectonics, geological, and hydro-geochemical studies indicated high geothermal potential of the study area (3S Kale, Şener et al.^2^, Bilim et al.^3^).

### Structural geology

The regional structural geology (Fig. 1) is governed by the subduction of the African Plate towards North beneath the Anatolian plate resulting in tectonic, volcanic, and seismic activity and an E-W oriented extension^4–7^. In this regime, NW–SE striking faults operate as dextral, and NE-SW trending faults as sinistral. Therefore, the TGF is a SW dipping fault with oblique-dextral slip, and the EF with a pure sinistral movement^8^.

Two smaller faults, sub-parallel to the regional set-up, are the Keçiboyduran-Melendiz dextral strike-slip fault (KMF) in the Ihlara Valley and the dextral strike-slip Göllüdağ Fault (GF). The latter is mostly buried beneath volcanic cones SE of Narlı Lake^9^. Normal faults striking WNW and NNW were observed in the vicinity of Narlı Lake, cross-cutting with a fault running NE-SW at the southern shore of the lake^10,11^ (Fig. 1B). A NE striking normal fault also crosses the Ihlara Valley near Ziga^11^. Further faults are mentioned by Burcak^12,13^; Şener et al.^2^; Doğan et al.^14^, but without referring to a certain stress regime or fault type. Thus, the regional structural setting of the study area is well understood while the local interconnection of the fault systems is not yet uncovered.

The regional geology is dominated by volcanic activity of Early Pliocene to Quaternary age at the larger Hasan, Keçiboyduran, and Melendiz volcano and several smaller volcanoes, cinder cones, and craters^9^. The crater of Narlı Lake is composed of pyroclastic rocks and lava flows of basaltic or andesitic composition of Holocene age with the latest activity 12 to 40 ka ago^10,15^. Andesitic, basaltic and rhyolitic rocks are the predominant lithologies in the study area, which locally interfinger with lacustrine sediments^2,16^. A Paleozoic marble exposure and overlaying Quaternary travertines outcrop at the northern edge of the Ihlara Valley, where they are still actively deposited by the Ziga springs^16,17^. The travertine exposes N-S oriented open fissures^14,17,18^. A smaller travertine zone is reported by Gevrek and Kazanci^10^ SW of Narlı Lake.

Geological layers exposed by drilling are basalt, rhyolite, andesite, and two limestone layers underlain by a basement of schist and gneiss^12^. The depth of the basement (1.5–3 km) and the regional heat flow suggest the largest plutonic intrusion in Central Anatolia (Upper Cretaceous) beneath the NE part of our study area, linked with an increased geothermal gradient^3^. Indeed, two shallow wells (220 m) in the Ihlara Valley show geothermal gradients of > 100 °C/km, with increased values in limestones^19^ and the first deep geothermal well at Sivrihisar has a gradient of 77 °C/km (Fig. 1B, Table 1, 3S Kale).

**Table 1:** Measured and estimated geothermal gradient in the study area using well data and K/Mg geothermometry.

Well/hot spring name see also Appendix)	Depth (km)	K/Mg geothermometer temperature (°C)	Geothermal gradient (°C/km)
MTA2 (well)	1.25	100	80
ACG3 (spring)	1.25	109	87
ZSMS (spring)	1.25	113	90
YSMS (spring)	1.25	106	85
AZ-1 (spring)	1.25	118	95
ZG-1 (spring)	1.25	106	85
ZG-2 (spring)	1.25	118	94
Sivrihisar (well, 3S Kale, measured data)	1.25	96 (295°C measured at 3.85km)	77

Water discharges in crater lakes at various locations in the study area, with Narlı Lake as the most prominent. Smaller freshwater lakes occur at the feet of several mountains. The largest river is the Melendiz River, flowing through the Ihlara Valley, following the KMF fault. Hot and warm springs appear throughout the whole study area (Fig. 1C, Appendix Figure 1).

## Results

### Bathymetric data

**Figure 2:**
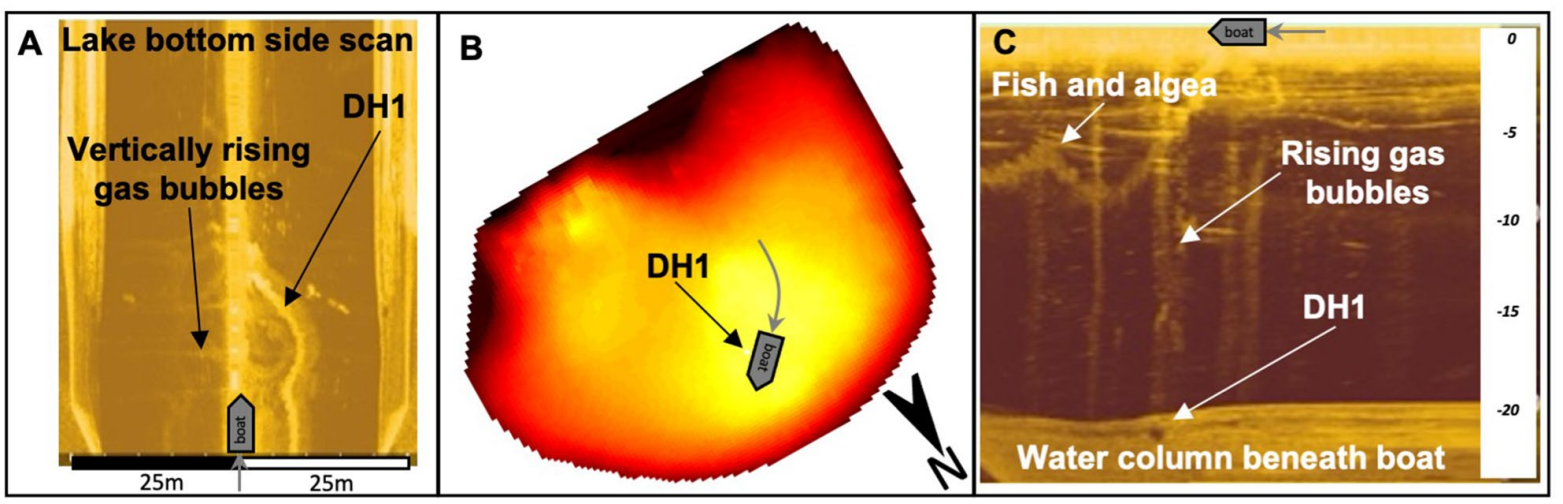
Sonar side scan images from the deep hole DH1, (**A**) Lake bottom in top view from the boat with a side-scan swath of 25 m. (**B**) Track of the boat. (**C**) Water column in profile view beneath the boat above deep hole DH1 with rising gas bubbles.

Bathymetry data from Narlı Lake show an average lake depth of 15 m, a major and a minor depression in the NW and SE of the lake, and two steep holes with 18 and 25 m depth (Fig. 1D,F). These holes (DH1 and DH2) are narrow cone-shaped openings with a diameter of 10 to 20 m, continuing vertically into the underlying bedrock. The sonar images of the holes show gas discharge from the hole (Fig. 2). The depressions around the holes are elliptically shaped in NE-SW direction. They present extensional basins between the overstepping dextral fault, intersected by thrust faults (Fig. 1D). Thus, the regional structural set-up is reproduced locally on the scale of Narlı Lake. The major dextral GF enters the lake in the SE and continues with a right-stepover before exiting the lake towards NW. The right-stepover forms an extensional basin causing the depressions in the lake (Fig. 1D). Additionally, two NE-trending thrust faults enter the lake in the S and intersect with the GF exactly at the deep hole locations (Fig. 1D). These faults also form topographic steps in the crater rim (Fig. 1E) and belong to a larger thrust fault setting due to the left-stepover of the GF to the KMF (Fig. 1A). The intersection of faults in the lake creates a weaker tectonic area allowing deep fluids to rise through the holes.

### Hydrogeochemical data

**Figure 3:**
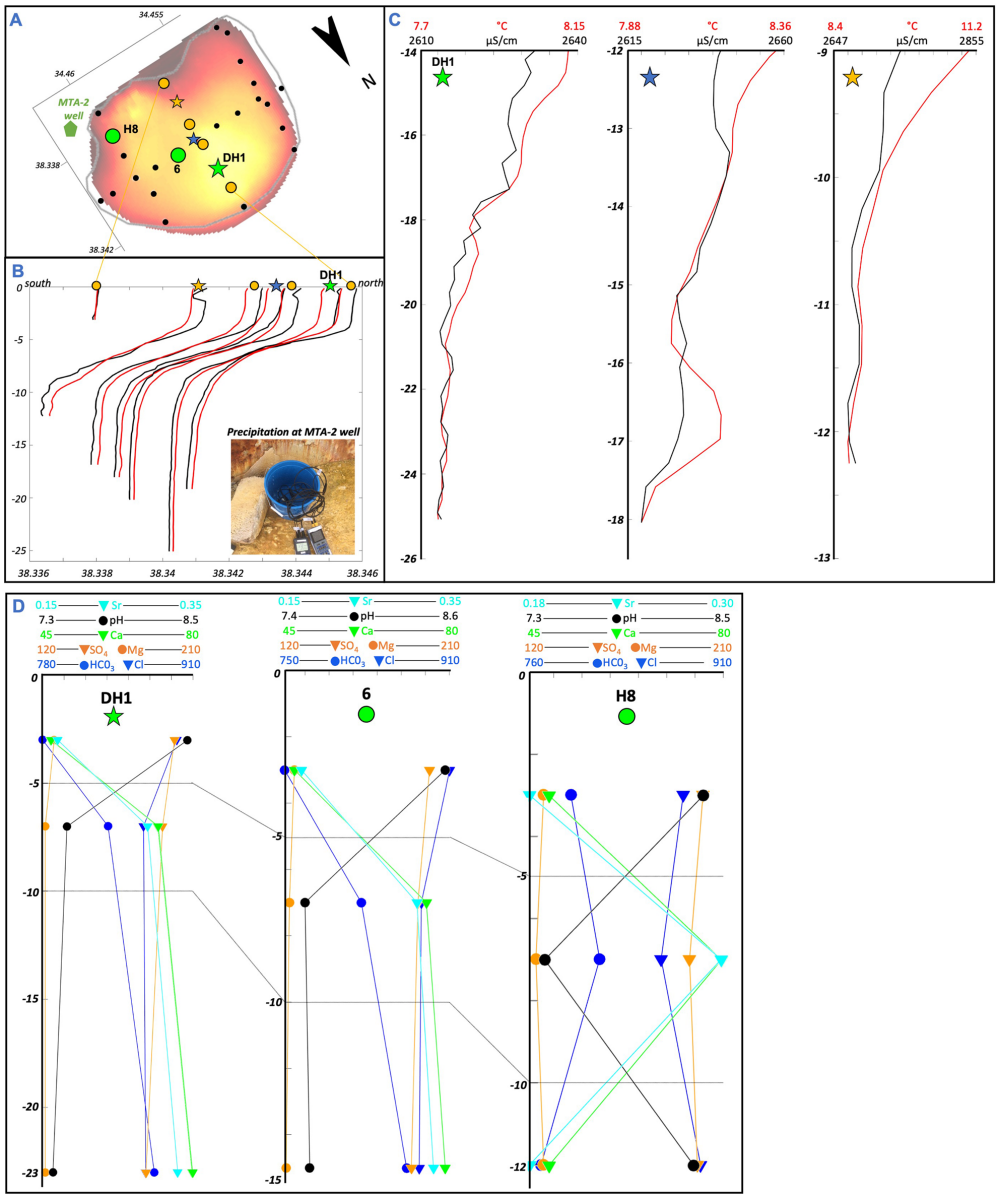
Geochemical signature of lake water. (**A**) Location of presented measurements along central N-S profile (orange dots and stars) and selected fluid sample locations (DH1, 6, H8). (**B**) Temperature (T) and electrical conductivity (EC) measurements over depth along the N-S profile (orange dots and stars). Insert: White-yellowish precipitation around MTA-2 well at the lake shore (location in **A**). (**C**) T and EC values in the deepest layer at three selected locations along the N-S profile showing parallel trends and peaks of T and EC. (**D**) Element concentrations and pH value over depth in all layers at three selected sample locations (DH1, 6, H8).

Profiles of temperature (T) and electrical conductivity (EC) have been measured at 34 locations in the lake and fluids have been sampled at three depths at DH1, 6, and H8 on a NE-SW trending profile (at 3 m, 7 m, and at maximum depth, representing the horizontal layering, Fig. 3). H8 represents an average water composition at a shallower depth, DH1 is the deepest hole and sample 6 is from the deeper middle part of the lake.

The profiles show three horizontal layers in Narlı Lake (Fig. 3B): from 0 to 5 m, 5 to 10 m, and 10 to 25 m depth, at which transition T and EC change rapidly. The layering might represent or be intensified by a seasonal thermal stratification. In general, T and EC reduce in parallel over depth. Peaks in temperature are also seen in electrical conductivity especially close to the lake bottom, although already temperature corrected (Fig. 3C). Electrical conductivity trends show more distinct peaks than temperature, especially at DH1. The most distinct peaks in DH1 are at 17.5 m, 21.5 m, and 23.5 m showing an increase of 5–10 µS/cm and ~ 0.05 °C. At the ‘blue star’ location the peaks are at a depth of 13.5 m and 16.5 m and represent an increase in EC of 5–8 µS/cm and ~ 0.15 °C (Fig. 3C). These variations could indicate a local warmer and saline water inflow through the lake bottom from surrounding rock formations.

The average lake water composition is dominated by Cl, HCO_3_ and SO_4_ as major anions and Na, K, Mg, Ca, Si and Sr as major cations at the following concentrations (Appendix Table 1): Cl (907 mg/l), HCO_3_ (770 mg/l), SO_4_ (208 mg/l) and Na (490 mg/l), K (180 mg/l), Mg (127 mg/l), Ca (47 mg/l), Si (49 mg/l) and Sr (0.2 mg/l). It has a pH of 8.5, a temperature of 22.6 °C, and electrical conductivity of 3483µS/cm. Concentrations of HCO_3_, Cl, SO_4_, Ca, Mg, Sr and the pH value also change over depth, while Si remains stable (Fig. 3D). The average trend in the lake, here represented by H8, shows an increasing trend of HCO_3_, Ca, and Sr and decreasing concentrations of Cl, SO_4_, Mg, and pH in the first two layers. This trend reverses in the deepest layer. At location 6 and the deep hole DH1, representing the deeper parts of the lake HCO_3_, Ca, and Sr increase over depth, while Cl, SO_4_, Mg, and pH decrease in concentration.

Fluid discharging from the MTA-2 well at the lakeshore (Fig. 3A) shows higher element concentrations compared to the lake water, especially for HCO_3_ (1524 mg/l), Ca (388 mg/l) and Sr (1.1 mg/l) but lower concentrations for Cl (423 mg/l), Si (28 mg/l), Na (236 mg/l) and Mg (46 mg/l). It has a slightly higher salinity and a slightly lower pH. Water constantly leaking from the wellhead has created a layer of white-yellowish precipitated minerals (insert in Fig. 3B).

**Figure 4:**
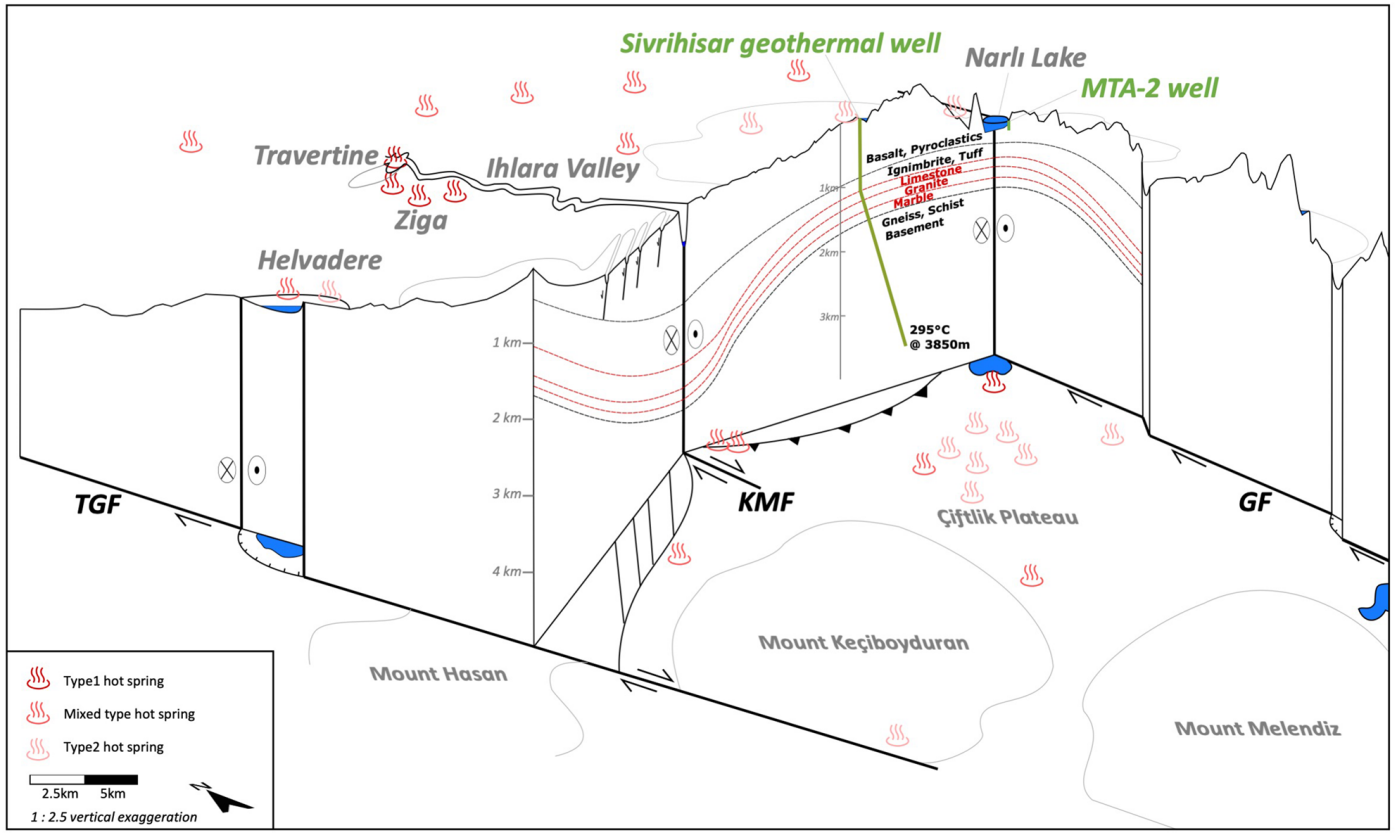
Sketch of structural-geological setting according to Fig. 1 with subsurface data from Burcak^12^. Suggested geothermal reservoir in limestone, granite and marble (red) and hot spring types.

Hot spring water in the study area has mainly two endmembers (Type 1: dark pink and Type 2: light pink, Appendix Table 1), while some hot springs show a mixed signature. The main distinction between Type 1 and Type 2 is a higher salinity and higher temperatures in Type 1. The by one-magnitude higher salinity is due to increased values of nearly all elements, but especially Cl, HCO_3_, SO_4_, Na, Ca, Mg, and K. Si and pH are slightly lower compared to Type 2 hot springs. Type 1 discharges from hot springs at Narlı Lake and from the Ziga springs along the Ihlara Valley (Figs. 1B, 4, Appendix Figure 1). Type 2 waters arise in hot springs near Sivrihisar, on the Çiftlik Plateau, and in Helvadere (Figs. 1B, 4, Appendix Figure 1). Intermediate mixed-types arise at the N edge of the study area, in the central study area, and in the SW study area (Figs. 1C, 4, Appendix Figure 1).

## Discussion

The combined approach of bathymetry and hydrochemical measurements allows a detailed understanding of geofluid-pathways and conclusions on the geothermal potential in the study area. It also adds details to the known structural geological set-up on a smaller scale.

Compared to former bathymetry studies in Narlı Lake^20,21^ our high-resolution data uncovered the deep holes and the fault indications in the lake. Previously known faults onshore around the lake can now be traced into the lake and attributed to a fault type related to the stress regime and fluid discharge. The small-scale fault set-up in Narlı Lake translates well to the regional model of the whole study area: Water discharges and deep holes occur where right-lateral NW trending faults intersect with perpendicular faults, especially at right stepovers. Here, the NW trending faults cause extension. Hence, we see four extensional areas including fluid discharge in the study area: within Narlı Lake at small-scale, at Helvadere (mid-scale), between Mount Hasan and Ihlara Valley, and along the southern GF at a larger scale (Figs. 1, 4). A large-scale compressional zone appears between the southern Ihlara Valley and Narlı Lake (Figs. 1, 4).

Fluid characteristics in the study area have been studied in lake water, hot spring water, and samples from wells. The hottest and most saline waters discharge from Type 1 hot springs and from the well close to the lake. Also, the lake water shows an increased electrical conductivity. These increased values are due to high Cl, HCO_3_, Na, and Ca concentrations, while Cl and Na dominate the lake water and HCO_3_ and Ca are predominant in hot springs and the well. All discharging water show similar high concentrations of Si. These high salinities and temperatures with increased Si concentrations are indicators of a geothermal influence on the system. High HCO_3_ and Ca concentrations suggest a contact of the fluids with limestone, while increased Cl, Na and Si show the strong influence of volcanic rocks. Also SO_4_, Mg, and K come probably from water–rock-interaction with volcanic formations. In summary, the geothermal fluid signature in this study area contains increased Cl, HCO_3_, Na, Ca concentrations and higher amounts of SO_4_, Mg, and K together with a low pH (Type 1). The more diluted Type 2 water and those with element concentrations between Type 1 and Type 2 also derive their water composition from deeper geothermal fluids but mix during ascension with groundwater at a shallower depth.

Fluids at the bottom of Narlı Lake show a water composition close to the geothermal signature (Type 1), with increased HCO_3_, Ca, and Sr, while the pH is lower at depth. Also, bubbles are observed close to the holes indicating gas discharge (most probable CO_2_ or H_2_S) from rock layers beneath the lake. At depth, where the gas is still partly dissolved, the pH is lower. Gas then diffuses in the lake during rise to the surface. This causes an increase in pH and a decrease in HCO_3_, Ca, and Sr, which precipitate in calcite or strontianite minerals. These minerals have an increased potential for supersaturation of 60–95%, as shown by hydrogeochemical models. Calcite or strontianite minerals are also seen in lake sediments of Narlı Lake^20,22,23^ and in white precipitation observed at the lakeshore and around the MTA-2 well (MTA: Maden Tetkik ve Arama Genel Müdürlügü, Öcal^24^; Bilen^15^; Dean et al.^23^). A similar phenomenon at a larger scale is observed at the Ziga springs, where massive travertine precipitation occurs with increased Ca and Sr contents ^17^. In the lake, the discharging H_2_S is oxidized on its rise to the surface causing the forming of SO_4_, as often observed in acidic volcanic lakes^25^. Increased Cl and Na concentrations at the lake surface are most probably due to surface evaporation from the lake body.

Based on this signature, we suggest that the geothermal reservoir in our study area is made out of volcanic and carbonatic rocks. Their intercalation is described in stratigraphic columns^12^ and reflects in our water signature. The volcanic rocks are rich in Cl, SO_4_, Mg, Na, K, and Si, while the local limestones mainly consist of HCO_3_, Ca, and Sr. Compared to volcanic lakes with neutral pH at other locations, our data show extremely high Cl, SO_4_, Na and increased HCO_3_, Ca and K concentrations^26^. This indicates a stronger fluid-rock interaction and a strong influence of limestones. The neutral pH in Narlı Lake indicates neutralization of acidic water by volcanic rocks at depth^27^.

The reservoir layers are located at 1 to 1.5 km depth and described as Marble, Granite, and Limestone overlain by Ignimbrite and Tuff^12^ (Fig. 4). The temperature in these layers is 96 to 118 °C, based on geothermal gradients in surrounding wells and on geothermometer calculations at springs (Table 1). Theoretically, hotter geothermal resources might exist in the basement rocks, but fluid pathways are most probably reduced to small or tight fractures. However, these fluid pathways can be enhanced at fault intersection points or extensional areas leading to an increase in permeability and therewith geothermal productivity. Therefore, geothermal well placement should always consider the structural set-up of the area.

## Conclusion and conceptual model

The successful placement of geothermal wells depends on targeting fluid-pathways in hot rock. Here, fluid flow mainly happens along faults and fractures. The origin and flow pathways of the fluids are preserved in their hydrochemical properties. Hence, the combination of high-resolution structural geological and hydrochemical data allow to guide geothermal potential estimation and well targeting.

This study shows that hot geothermal fluids discharge in our study area at strike-slip fault intersections with thrust or normal faults (Narlı Lake, Ziga Springs, Helvadere). At these tectonically active intersection points, fluids find pathways to percolate upwards. In contrast to these favorable environments, fluids rising in less faulted/fractured areas show a mixed signature of reservoir water and shallow groundwater. The detailed hydrochemical composition of fluids allows us drawing conclusions on the reservoir rock composition and depth. In our case, the reservoir rock is a mixture of limestones and volcanic rocks with high concentrations of Cl, HCO_3_, SO_4_, Na, Ca, Si and Sr at a depth of 1 to 1.5 km.

Future studies in this area would benefit from re-collecting samples from the presented locations supplemented by isotopic analyses, which are able to give information on evaporation rates and volcanic gas input into the lake^28^. Furthermore, detailed gas analysis of the subaqueous rising bubbles could reveal their type and origin, which is relevant for chemical and volcanic reactions in the lake^29^.

The approach presented shows that it is beneficial to integrate a structural-geological set-up, fluid pathways, and hydro-chemical fluid properties to understand the geothermal potential at volcanic lakes. Detailed lake studies add important information and give direct access to the sub-surface structures and composition due to the preservation of geological features at mostly uneroded lake bottoms. Therefore, such an approach is suggested to be used for geothermal exploration and well targeting if lakes are present.

## Methods

### Bathymetry

We measured bathymetry from a boat going in spirals on Narlı Lake with decreasing diameter towards the lake center. The instrument used was a Lowrance Elite TI 7 from Navico including CHIRP sonar, GPS navigation, and a multibeam echosounder with side-scan and down-scan imaging tool capturing structures at lake bottom including gas discharge (Fig. 2). Data have been processed and transformed by Kriging analysis using software from ReefMaster Ltd and self-written Matlab codes. Processed depth data achieve an accuracy of 0.5 m in 2D and 3D (Fig. 1D,F).

### Hydrogeochemistry

Hydrochemical depth profiles have been logged using the CastAway-CTD logger from SonTek including conductivity, density, temperature, pressure, and GPS. It allows sampling at a 5 Hz sampling rate, translating to 15 cm sampling intervals. 34 depth profiles have been measured on several N-S and E-W profiles (Fig. 3A). Two to three repeat logs were run at the same spot to average out temporary fluctuations. Additionally, water samples have been taken at three locations indicated in Fig. 3A at three depths in the lake and additionally from a well at the lakeshore. Details on the sampling technique and analysis can be found in Brehme et al.^1,30,31^. The joint interpretation of our data with the literature on structural geology and hydrogeochemical properties of lakes and hot springs allows us deriving the link between geological structures and geofluid-pathways.

## Supplementary Information

Supplementary Information.

## Data Availability

Data and computer codes used to analyze the data in this study are available from the corresponding author on request.
