# Supplementary material for: Fluid pathways identified beneath Narlı Lake (Central Anatolia) show the geothermal potential of former volcanoes
Source: Sci Rep. 2021 Apr 22;11:8773. doi: 10.1038/s41598-021-87743-5 (PMC8062672; doi:10.1038/s41598-021-87743-5)
Supplement: Supplementary file 1 — Supplementary Information. [file 41598_2021_87743_MOESM1_ESM.docx]

Fluid pathways identified beneath Narlı Lake (Central Anatolia) show the geothermal potential of former volcanos

**Maren Brehme ^*1,2^, Ronny Giese^2^, Ugur Erdem Dokuz^3^ and Fatih Bulut^4^**

*^1^Delft University of Technology, Department of Geoscience and Engineering, Stevinweg 1, 2628CN Delft, Netherlands*

*^2^Helmholtz Centre Potsdam, GFZ German Research Centre for Geosciences, Geoenergy, Telegrafenberg, 14473, Potsdam, Germany*

*^3^Niğde Ömer Halisdemir University, Department of Geological Engineering, Faculty of Engineering, 51240, Niğde, Turkey*

*^4^Boğaziçi University, Kandilli Observatory and Earthquake Research Institute, Geodesy Department, 34684 Uskudar-Istanbul, Turkey*

*^*^ corresponding author: Maren Brehme, m.brehme@tudelft.nl*

Appendix Table 1: Hydrochemical properties of well, lake and hot spring samples from this study and literature (dark pink: Type1 waters, light pink: Type 2 waters, pink: mixed signature)

Appendix Figure 1: Names and location of springs from Table 1 in structural setting according to Figure 4
